# Nearly 200 years of sustained selection have not overcome the leaf area–stem size relationship in the poinsettia

**DOI:** 10.1111/eva.12634

**Published:** 2018-05-16

**Authors:** Laura Trejo, Julieta A. Rosell, Mark E. Olson

**Affiliations:** ^1^ Laboratorio Regional de Biodiversidad y Cultivo de Tejidos Vegetales Instituto de Biología, Universidad Nacional Autónoma de México México Mexico; ^2^ Laboratorio Nacional de Ciencias de la Sostenibilidad Instituto de Ecología Universidad Nacional Autónoma de México Ciudad de México Mexico; ^3^ Instituto de Biología Universidad Nacional Autónoma de México Ciudad de México Mexico

**Keywords:** adaptation, allometry, artificial selection, Constraint, corner's rules, domestication, *Euphorbia pulcherrima*, selection

## Abstract

Organismal parts often covary in their proportions, a phenomenon known as allometry. One way of exploring the causes of widespread allometric patterns is with artificial selection, to test whether or not it is possible to move populations into “empty” allometric space not occupied by the wild type. Domesticated organisms have been subject to many generations of selection, making them ideal model systems. We used the domesticated Christmas poinsettia *Euphorbia pulcherrima* in combination with wild populations to examine the origin of the proportionality between leaf area and stem size, which scales predictably across nearly all plants. In accordance with the stated aims of breeders to produce more compact plants, we predicted that domesticated poinsettias would have greater leaf area for a given stem volume than the tall, lanky wild ancestors. Our data rejected this prediction, showing instead that domesticates have leaf area–stem volume relationships identical to the wild ancestors. Presumably the metabolic dependence between stems and leaves makes the leaf area–stem volume relationship difficult to overcome. The relative fixity of this relationship leads to predictable covariation in other traits: The fuller outlines of domestic poinsettias involve significantly shorter internodes, and given a constant leaf area–stem volume relationship, smaller individual leaf areas. At the same time, domestic poinsettias are subject to selection favoring breakage resistance, which is achieved via thicker stems for a given length rather than stiffer stem tissue resistance to bending. Our results show that domesticated poinsettias differ from wild plants in a suite of traits including leaf size, internode distances, and stem length–diameter relations, but despite over 200 years of selection favoring rounded outlines, there has been no change in the total leaf area–stem volume relationship, helping to predict which changes are likely achievable and which will not be under continued artificial selection and in the wild.

## 1 INTRODUCTION

The evolution of morphological diversity is bounded by biophysical and adaptive limits, including biases imposed by resource allocation tradeoffs, adaptive covariation, or the dynamics of development (Fusco, 2001; West‐Eberhard, 2003; Arthur, 2004; Losos, 2011). Whether selection is artificial or natural, understanding the breadth of developmental possibility is essential to understand why some morphologies are observed and others are not (Drake & Klingenberg, 2010; Olson, 2012). Situations in which the commonly observed morphologies represent only a tiny subset of the developmentally possible are consistent with the action of selection. Alternatively, the common morphologies could be the only ones, or among the few ones, that are developmentally possible. This second scenario could represent a situation in which selection has extinguished most variation, or one in which variation is or even was never possible, a phenomenon often discussed under the umbrella term “constraint” (Pigliucci, 2007; Pigliucci & Kaplan, 2006; Richardson & Chipman, 2003; Sansom, 2009; Schlichting & Pigliucci, 1998). Such mapping of developmental potential (understood as which variants can be produced and which cannot or are not) is also important for domestication efforts because they can inform regarding morphologies that are likely to be achievable and which are likely difficult or impossible. Whatever the case, to identify the causes underlying developmental limits or biases must begin with a mapping of developmental potential (Olson, 2012).

For several reasons, domesticated organisms offer particularly attractive systems for exploring developmental limits and biases and their interaction with selection. One reason is that unlike in wild organisms, the features under selection are often well known and explicitly specified (Drake & Klingenberg, 2010). This means that it is possible to know what features breeders have attempted to accentuate or reduce, or which trait combinations are favored. In contrast, in wild populations it is often difficult to determine which attribute is actually under selection (MacColl, 2011). In addition, the wild ancestors of domesticated organisms are often living and can be compared directly to their descendants. The domesticates have often been under selection for hundreds if not thousands of years, and the possibility of comparing the products of such long periods of selection with their starting point is, in macroscopic organisms, found only in situations of domestication (Drake & Klingenberg, 2010; see also Frankino, Emlen, & Shingleton, 2010).

We use a well‐known domesticated plant, the Christmas poinsettia (*Euphorbia pulcherrima* Willd. ex Klotzsch), to investigate the causes of one of the most pervasive trait relationships across the woody plants, the leaf size–stem size relationship. This relationship includes the observation that worldwide, the total leaf area supported by a twig is predicted by the diameter of the twig supporting them (Westoby & Wright, 2003). This pattern, known as Corner's rules, is pervasive, having been documented across virtually all flowering plant lineages, on multiple continents, in vastly differing vegetation types and climates (Ackerly, 1996; Ackerly & Donoghue, 1998; White, 1983a). Corner's rules state that plants with large leaves have thick twigs and branch sparingly; plants with small leaves have thin twigs that branch intricately (Leslie, Beaulieu, Crane, & Donoghue, 2014; Sun, Jin, & Shi, 2006; Westoby, Falster, Moles, Vesk, & Wright, 2002; White, 1983b; Wright, Falster, Pickup, & Westoby, 2006). What causes Corner's rules is actively being elucidated (Smith, Sperry, & Adler, 2017), and documenting how plants fill leaf area–stem size space is essential in this effort.

One essential assumption regarding the cause of the leaf size–stem size spectrum has to do with the mutual metabolic dependence between leaves and stems. Leaves meet the metabolic needs of stems and roots, whereas stems mechanically support leaves and supply them with water. Presumably this metabolic proportionality drives the generally strong leaf area–stem size relationship (Olson, Aguirre‐Hernández, & Rosell, 2009). Stated in terms of developmental potential, the space that the leaf area–stem size relationship describes can be thought of as having three areas (see Olson, 2012; Olson & Arroyo‐Santos, 2015). The first is the leaf area–stem size scaling line that most plants fall along, which shows that plants with greater stem size have predictably higher leaf area. The area below the scaling line corresponds to plants that have less leaf area for a given stem size than most plants. Cacti and many other plants with small or even no leaves are clear examples that in the correct selective contexts, usually warm drylands, plants can have markedly lower leaf area for a given stem size (Eggli, 2002). In other words, the largely empty space beneath the leaf area–stem size scaling line is accessible developmentally but apparently favored by selection only in extreme situations. What is not so clear is whether the area above the line is accessible. This area corresponds to plants with more leaf area for a given stem size than typical plants have. Unlike the reduced leaf area below the scaling line, few or no examples have been offered of plants with relatively high leaf area. If plants can be found that fall above the scaling line, then they would suggest that plants with “too much” leaf area are developmentally accessible, that is, that these variants can be produced. If they can be produced, then it makes it likely that their rarity in the wild is due to selection acting against them in most situations. Finding, on the other hand, that they apparently cannot be produced would open the door to study of the cause of this inaccessibility and would potentially modify the adaptive interpretation of the leaf size–stem size spectrum. Because these alternative scenarios are very different, it is therefore very important to explore the accessibility of the area above the leaf area–stem diameter scaling line.

**Figure 1:**
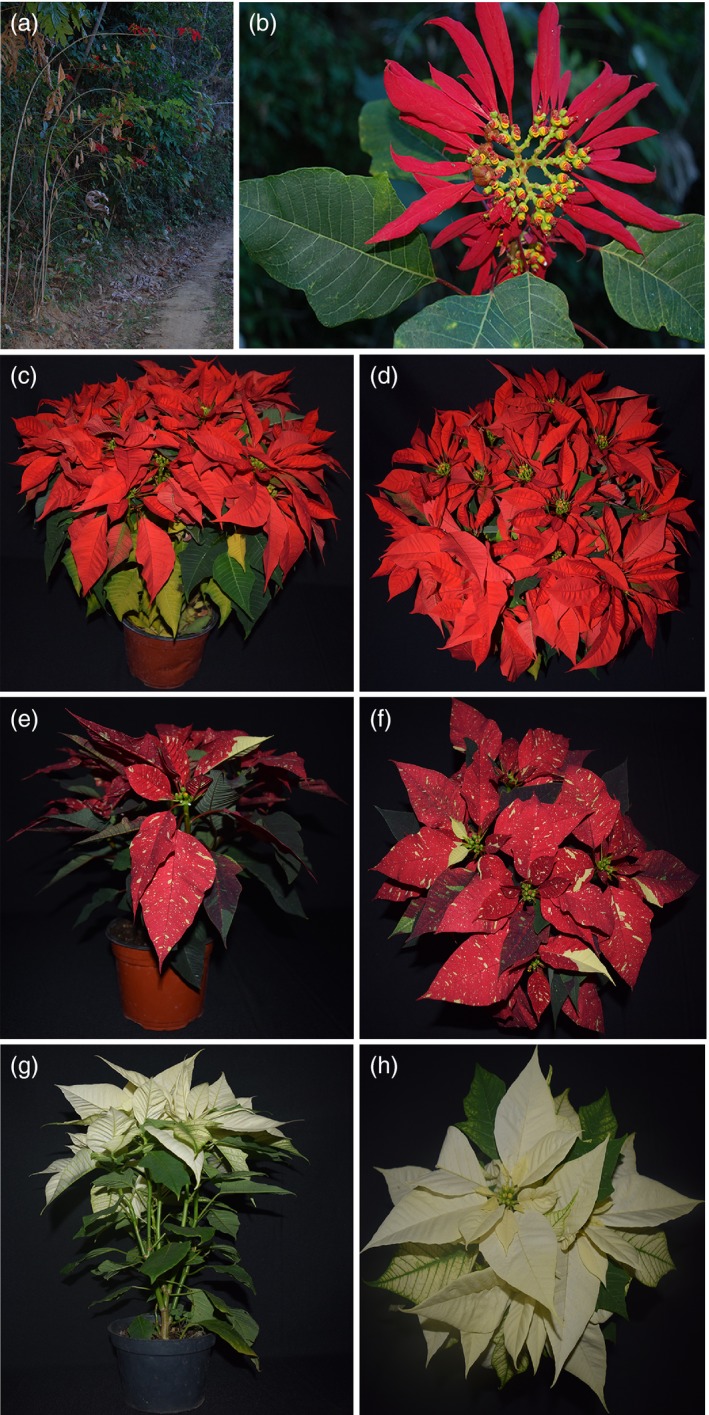
Habit differences between wild and domesticated poinsettias. (a, b) Wild population near Concordia, Oaxaca. (a) Plant habit, showing long, sparingly branched, lanky stems 3 m tall. (b) Wild inflorescence, showing narrow bracts and abundant floral structures at the center. (c–h) Domesticated poinsettias to show the full, rounded outlines of cultivated plants, contrasting with the lanky wild plants. (c, d) Orange Spice, showing the very full outline with little empty space between leaves; (e, f) Red Glitter, showing the very wide leaves and bracts; (g, h) Polar Bear, showing abundant branching from the base

The poinsettia is ideal for examining whether leaf area can be increased for a given stem size because, in the stated goals of breeders, it has apparently been under selection in exactly this direction for nearly two centuries (Ecke, Matkin, & Hartley, 1990; Kobayashi, 2012; Lee et al., 1997; Taylor, López, Currey, & Janick, 2011). Wild poinsettias are lanky shrubs or small trees in mid‐elevation tropical subdeciduous forests of the Mexican tropical Pacific slope with cane‐like stems to over 6 meters long, mostly unbranched and clothed with distantly spaced leaves (Trejo et al., 2012; Figure 1). A rich historical record documents with illustrations the transformation from the lanky wild ancestor to the dense, rounded profile of the domesticates, as well as recording the aims of the breeders. The earliest efforts selected for, among other characteristics, homeotic replacement of the flower heads for colorful bracts, and shortened inflorescence internodes (e.g., Graham, 1836; Navarro, 1992). Starting with the still tall and lanky early cultivars (Ecke et al., 1990; Taylor et al., 2011), poinsettia breeding has been directed at creating smaller, compact plants with more rounded outlines with a bushy habit (Kobayashi, 2012; also Taylor et al., 2011; Lee et al., 1997). As part of this program of shortening and creating a rounded profile, any heritable variant with short internodes and relatively high leaf area for a given stem size would be seized upon and accentuated. This well‐documented vector of domestication makes the poinsettia a particularly fertile system in which to look for deviation from the common global leaf size–stem size relationship.

Changes in biological shape or size, such as the leaf area–stem size allometric relationship, also inevitably involve changes in the mechanical behavior of a structure (Rosell et al. 2012). Therefore, it is essential to understand how any differences in scaling between wild and domestic poinsettias could be associated with differences in the mechanical properties of their stems. We examine here the Young's modulus of the stem *E*_stem,_ which is an index of how well the material that makes up a given structure resists deformation (Niklas, 1992; Vincent, 1992). Examining stem mechanical properties in poinsettia is likely to be informative because one of the well‐documented vectors of selection of poinsettia breeders is to increase resistance to stem breakage during shipping (Ecke, 2011; Taylor et al., 2011). As a result, it seems possible that selection has altered stem mechanical properties in domesticates with respect to the wild ancestor in ways that might be linked to leaf area–stem diameter scaling.

We collected material from wild populations of poinsettia and compared the leaf size–stem size scaling patterns with morphologically divergent cultivars. Given this range, we fully expected to find marked deviation in leaf size–stem size scaling consistent with movement into the “too much leaf” zone. We found instead that while domestic poinsettias have smaller leaves, shorter internodes, and thicker stems than wild poinsettias, the leaf area–stem relationship remains identical between the two. We discuss reasons for why the leaf–stem relationship should be so difficult to move into the “too much leaf” area and how this fundamental relationship results in predictable patterns of covariation in other traits.

## 2 METHODS

### 2.1 Leaf area–stem volume scaling

Previous phylogeographic work (Trejo, Olson, & Bye, 2015; Trejo et al., 2012) mapped the relationships between wild and domesticated poinsettias. We used these data to select wild populations closely as well as distantly related to the modern cultivars. We sampled from the two largest wild poinsettia populations that we have been able to locate. One was from La Calera canyon in the Municipio (County) of Casimiro Castillo, in the southwestern part of Jalisco State in southwestern tropical Mexico, at 19°40.902′N, 104°25.035′W and an elevation of about 800 m. a. s. l. The vegetation is tall tropical subdeciduous forest. The other population was on steep slopes near Finca La Concordia on Cerro Espino mountain, Pochutla District, in southern Oaxaca State, at 15°52.652′N, 96°25.869′W and an elevation of about 620 m. a. s. l., again in tall semideciduous tropical forest. To maximize chances of finding departures from wild‐type leaf–stem proportionality, we selected three of the main current and morphologically divergent cultivars, which in their commercial descriptions are described as spanning a range of leaf densities (Ecke, 2011). The cultivar Orange Spice is described as having a relatively sparse complement of narrow leaves. Red Glitter is described as having a rounded, compact outline, and a high density of wide leaves. Finally, Polar Bear is noted as being similar to Red Glitter but more highly branched (Figure 1). In all, we sampled one branch of each of 16–34 individuals per wild population or variety for a total of 118 individuals.

We sampled a range of stem diameters across populations and varieties to provide the necessary variation for fitting allometric models. We collected one branch per individual, measured its basal diameter fresh using digital calipers, labeled each leaf and bract, and pressed and dried the leaves and bracts in a herbarium press. In the laboratory, we measured leaf area, including bracts and petioles, using the program WinFolia Basic V2011a (Regent Instruments Inc 2011, Quebec, Canada). Noting the number of leaves per stem, we then summed the leaf area per branch. We represented stem volume using the basal diameter, stem length, and the formula for the volume of a cylinder. These data are available in Table S1.

We then fit multiple mixed‐effects linear models predicting per‐branch total leaf area (Bolker et al., 2009). We started with a model with the fixed effects log_10_ stem volume, a binary cultivated/wild categorical variable, and a log_10_ stem volume wild/cultivated interaction term. The random effect included the categorical variable population/variety to which the branch (and individual) belonged and had five levels (Concordia, La Calera, Orange Spice, Polar Bear, and Red Glitter). This random effect took into account that branches (and individuals) were nested within populations/varieties. This first model allowed the populations/varieties to have a random intercept. We then fit another model allowing population/varieties to have a random slope in addition to a random intercept. We compared these two models (random intercept vs. random slope and intercept) using likelihood ratio tests and the Akaike Information Criterion (Zuur, Ieno, Walker, Saveliev, & Smith, 2009). Once the best model was chosen based on the random component, we examined whether cultivated and wild individuals differed in their leaf area–stem volume allometry testing the significance of the log_10_ stem volume wild/cultivated interaction term. When this term was nonsignificant, we refit the model without it. Lack of significance of the interaction term indicated that the leaf area–stem volume scaling had a similar slope between wild and cultivated plants. This also meant that we could check straightforwardly for differences in leaf area–stem volume scaling through the intercept of the fixed component and directly address our central question of whether cultivated plants have higher leaf area for a given stem volume than do wild plants (i.e., higher leaf area–stem volume intercepts). When the cultivated/wild categorical variable was nonsignificant, the model suggested no intercept difference between provenances and the model was refit without this term. We verified homoscedasticity and normality of data using graphical tools. We fit all models using the R package nlme (Pinheiro, Bates, DebRoy, & Sarkar, 2017) and calculated R^2^ (Johnson, 2014; Nakagawa & Schielzeth, 2013) with the R package r2glmm (Jaeger, 2017) using R v.3.4.0 (R Development Core Team, 2017).

### 2.2 Mean individual leaf area–stem volume scaling

To ask whether selection for shorter, more rounded poinsettias has affected the mean size of individual leaves (as opposed to the total leaf area supported by stems), we followed a similar procedure as for total leaf area per branch. Using the data in Table S1, we calculated the mean area of leaves per branch and fit a model predicting log_10_ mean individual leaf area based on log_10_ stem volume, a binary cultivated/wild categorical variable, and a log_10_ stem volume wild/cultivated interaction term. As for the previous model, the random component included the categorical variable population/variety to which the branch belonged (the same levels as for models predicting total leaf area). We again compared random intercept versus random slope and intercept models. Once the best random component was identified, we tested the significance of the interaction term.

### 2.3 Stem biomechanics

To test the notion that the domestication of poinsettia might involve differences in stem mechanical properties, we gathered samples from the large wild population at La Calera, and from Red Glitter and Polar Bear cultivars. The mechanical tests were conducted in the season subsequent to leaf area–stem diameter allometric measures, and the wild population of Concordia and the domestic cultivar Orange Spice were not available. We replaced these with a wild population near the town of Taxco in Guerrero State, one of the wild populations most closely related to domesticates, and with a similar cultivar to Orange Spice known as Subjibi. In total, we gathered mechanical data from 37 segments from domesticates, and 48 segments from wild plants for a total of 85 segments from 47 individuals.

For most individuals, we selected two stems per plant for mechanical testing. However, for 11 wild individuals (83%) only one segment was available. Wild plants often consisted of single stems, so when these were sufficiently long, we separated them into two segments for testing. In the case of domesticates, we selected two stems per individual, which given their short lengths yielded a single test segment per stem. We cut segments 20–35 cm in length, to provide a 1:20 diameter: length ratio to minimize shear (Vincent, 1992). We tested the segments in three‐point bending with an Instron 3345 mechanical test machine equipped with a 5‐kN load cell. Stem Young's moduli (*E*_stem_) were calculated using Instron System IX/s software (Instron Corporation, Canton, MA), using the basal diameter of the tested segment in the calculation of the second moment of area *I* (Niklas, 1992). Poinsettia stems have hollow piths, but we calculated *I* as though the stems were solid; this procedure underestimates the absolute *E*_stem_ but the results are entirely comparable between individuals and segments. The mechanical demands made on a given stem segment are largely described by how far the segment is from the stem tip, because this distance is directly related to the bending moment that the segment is subjected to. Therefore, we measured the distance to the stem tip from the midpoint of our test segments and used this distance as the metric of comparison between wild and domesticated poinsettia mechanical properties. These data are given in Table S2.

To examine the scaling of stem stiffness with distance from the tip, we used mixed‐effects linear models following a similar procedure as previously explained. We predicted log_10_ *E*_struct_ based on log_10_ distance from the tip, a binary cultivated/wild categorical variable, and a log_10_ distance from the tip wild/cultivated interaction term. The random component of this model included the individual to which the segment belonged nested within the population/variety (with five levels, Guerrero, La Calera, Polar Bear, Red Glitter, and Subjibi) to which the individual belonged. We followed the same procedure to select the best final model.

### 2.4 Internode length–stem volume scaling

Plants with denser outlines could be achieved by selection favoring shorter internodes. If stem dimensions and mean individual leaf area remain unchanged, shorter internodes imply greater total leaf area. When preliminary data suggested that domesticates bear slightly less rather than more total leaf area than similar‐sized stems of wild plants, we tested for differences in internode lengths between wild and domestic plants, to test the possibility that denser‐appearing domesticates are achieved via changes in internode length rather than in total leaf area. We collected wild plants from the La Calera population, which is distantly related to the domestic poinsettia, as well as two populations close to Taxco in the area directly ancestral to the cultivated poinsettia, at Casallas (18°33.360′N, 99°35.166′W, elevation 1630 m.a.s.l.) and La Anda (18°33.412′N, 99°57.511′W, elevation 800 m.a.s.l.), Guerrero State, as well as two populations in nearby Morelos State, Texcal (18°53.421′N, 99°08.328′W, elevation 1485 m.a.s.l.) and Amatlán (18°58.583′N, 99°02.019′W, elevation 1620 m.a.s.l.), also very closely related to the domesticates. We also sampled the cultivars Orange Spice and Red Glitter. Polar Bear was not commercially available during the sampling for internode length, so we substituted it with the very similar cultivar Lemon Snow. We measured stem volume and the distance between each internode on one branch per individual in 12–41 individuals from wild populations or varieties, for a total of 210 individuals (53 domestic, 157 wild) and 3849 internode distances. Data are given in Table S3.

To examine whether for a given stem volume domestic and wild poinsettias differ in their internode distances, we calculated a mean internode distance per branch (and per individual) and fit mixed‐effects linear models. In this case, we predicted log_10_ internode distance based on log_10_ stem volume, a binary cultivated/wild categorical variable, and a log_10_ stem volume wild/cultivated interaction term. As for the leaf area models, the random component of this model included the population/variety to which the branch (individual) belonged (with eight levels La Calera, Casallas, La Anda, Amatlán, Texcal, Orange Spice, Red Glitter, and Lemon Snow). As for previous models, we identified the best random component and tested the significance of the interaction term.

### 2.5 Stem length–stem diameter scaling

To examine whether domestic poinsettias have thicker stems for a given stem length, we predicted log_10_ stem length based on log_10_ stem diameter, a binary cultivated/wild categorical variable, and a log_10_ stem diameter wild/cultivated interaction term. The random component included the population/variety to which the branch (individual) belonged (same levels as models predicting leaf areas). We followed the same procedure as for previous models.

## 3 RESULTS

### 3.1 Total and individual leaf area–stem volume scaling

The 118 stems measured for testing our leaf area scaling hypotheses bore an average of 27.8 leaves per stem, ranging from 14 to 40 in stems of domestic individuals to 7 to 98 in those of wild plants. In domestic plants, stem diameter ranged from 2.5 to 8.0 mm, stem length from 51.4 to 420 mm, stem volume from 276.5 to 18,404 mm^3^, and area per leaf from 6.2 to 34.7 mm^2^, whereas in wild plants stem diameter ranged from 2.6 to 12.8 mm, stem length from 63.0 to 1510 mm, stem volume from 532.3 to 195,218.6 mm^3^, and area per leaf from 11.5 to 102.9 mm^2^.

**Figure 2:**
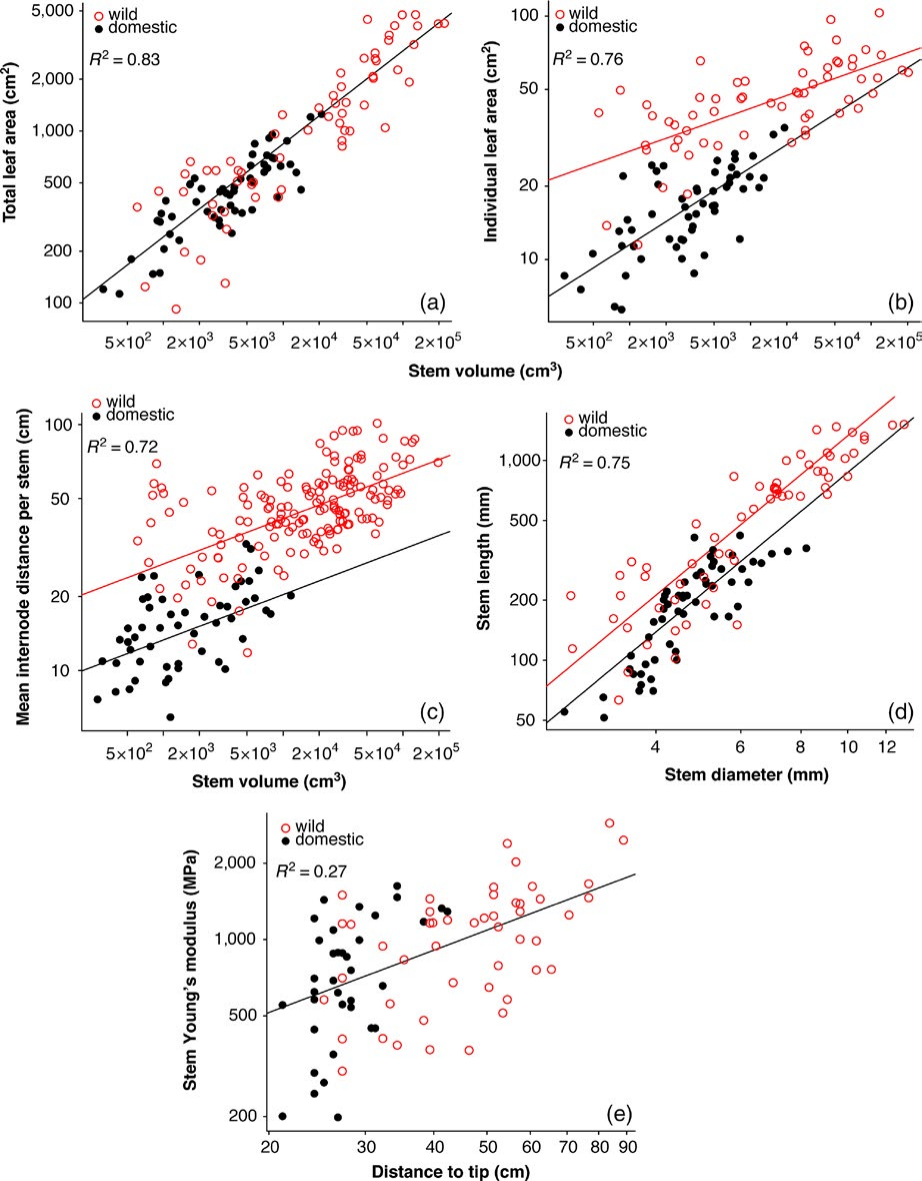
The constant total leaf area–stem volume relationship and its influence on trait relationships between domestic poinsettias and their wild ancestors. (a). Despite nearly 200 years of breeding small poinsettias with full outlines, the relationship between total leaf area and stem volume has not been altered with respect to the wild ancestor. (b) Although their total leaf areas are the same for a given stem volume, domesticated plants have smaller individual leaf areas. (c) Smaller individual leaf area with similar total leaf area requires more numerous leaves in the domesticate, an expectation borne out by our data, which show that for a given stem volume, domesticates have not only smaller leaves but more of them per unit of stem length, as indicated by their significantly shorter internodes. (d). Likely reflecting selection favoring breakage resistance, domesticates also have thicker branches for a given stem length, the only way that domesticates could resist breakage better than wild plants, given that their stem tissue mechanical properties do not differ (e)

Our first model tested for differences in log_10_ total leaf area for a given log_10_ stem volume between cultivated and wild plants, nesting individuals within populations/cultivars, the variable that was included as a random effect. In our initial model, the log_10_ stem volume cultivated/wild interaction term was nonsignificant (*p* = .126), indicating similar total leaf area–stem volume scaling between cultivated and wild plants (Table 1). We refit the model without the interaction term and examined whether cultivated and wild plants differed in intercept, which they did not (*p* = .570). With an *R* ^2^ of .83, the final model with just log_10_ stem volume and the cultivated/wild variable as fixed effects fit the data very well. The random component of this model indicated that populations/varieties had differences in intercepts but not in slopes (Table S4). Counter to our expectations, cultivated plants did not have a higher leaf area–stem volume intercept than the wild plants (Figure 2a). Instead, despite their markedly different overall appearances, and the much fuller outlines of cultivated as compared to wild plants (Figure 1), domesticated poinsettias have the same leaf area for a given stem volume as do the wild ancestors.

**Table 1:** Mixed‐effects models fit to test for differences in the allometry of wild and domestic poinsettias. Coefficients for fixed effects are shown with 95% confidence intervals in parentheses. All continuous variables were log_10_‐transformed. In all cases, the model with a random intercept had a better fit than the model with a random slope and a random intercept, except for the model predicting stem length (see Table S4)

	Total leaf area ~ stem volume + cultivated/wild	Individual leaf area ~ stem volume * cultivated/wild	Mean internode distance ~ stem diameter + cultivated/wild	Stem length ~ stem diameter + cultivated/wild	*E*_stem_ ~ distance to tip + cultivated/wild
Sample size	118	118	210	118	85
*R* ^2^	.83	.76	.72	.75	.27
Equality of slopes	*p *= .126	*p *= .004	*p *= .278	*p *= .769	*p *= .126
Equality of intercepts	*p *= .570	‐	*p *< .005	*p *= .043	*p *= .796
Cultivated intercept	0.761 (0.584, 0.937)	0.113 (−0.175, 0.401)	0.743 (0.629, 0.858)	0.934 (0.368, 1.500)	1.649 (1.097, 2.201)
Wild intercept		0.917 (0.035, 1.799)	1.052 (0.801, 1.304)	1.118 (0.378, 1.857)	
Cultivated slope	0.541 (0.495, 0.587)	0.316 (0.237, 0.395)	0.124 (0.100, 0.147)	2.006 (1.161, 2.850)	0.817 (0.463, 1.171)
Wild slope		0.176 (0.003, 0.349)			
Figure	2a	2b	2c	2d	2e

Our subsequent model tested for differences in log_10_ mean individual leaf area controlling for log_10_ stem volume among cultivated and wild individuals. This model also fit the data very well with an *R* ^2^ of .76. The model suggested differences in slopes across populations (*p* = .004; Table 1). Although this slope difference precludes a direct comparison of intercepts to test our hypothesis, Figure 2b shows that fitted lines do not intersect and that the wild individuals tend to have larger leaves than cultivated ones along the sampled range, which includes most of the size range for these plants. The random component of the model suggested that populations/varieties differed in intercepts but not in slopes as in the fixed component. In summary, although total leaf area does not differ between domestic and wild plants, domestic plants have smaller leaves, and therefore should have shorter internodes, a prediction we then tested.

### 3.2 Internode length–stem volume scaling

Finding similar leaf area–stem volume relations but differing leaf sizes between wild and cultivated plants suggested that the stems of domesticated plants are more densely clothed in leaves. This difference should be reflected in shorter internodes in domestic plants, consistent with our results. The 210 stems measured bore an average of 18.3 internodes per stem, ranging from 3 to 28 in domestic individuals and from 2 to 64 in wild ones. Internode distances varied from 6.3 to 32.1 mm in domestic stems, with a mean of 14.7, to 11.6–99.6 mm in wild ones, which had a mean of 47.8.

Our model showed that internode distance scaled with stem volume with an *R* ^2 ^= .72, with larger stems having longer internodes. This scaling occurred at the same rate between domestic and wild plants (nonsignificant log_10_ stem volume cultivated/wild interaction, *p* = .278; Table 1), permitting straightforward comparison of intercepts between wild and cultivated plants. For a given stem volume, internode distance in cultivated plants is significantly shorter than in wild ones (Figure 2c). In other words, for a similar plant size, domesticated poinsettias have significantly shorter internodes than wild plants, although the total leaf area that domestic plants support is the same as wild plants for a given stem volume. Again, the random component of the model suggested a similar behavior of similar slope and differing intercepts within populations/varieties (4).

### 3.3 Stem length–stem diameter scaling

Wild poinsettias have more closely spaced leaves than wild plants, contributing to their fuller outline, but they also have smaller leaves. Presumably there is a metabolic dependence between leaf area and the volume of metabolically active tissue within the stem. Given a constant leaf–stem metabolic relationship, any change in total leaf area should be reflected in a concomitant change in stem volume. Changes in stem volume can be accomplished by changes in stem length or diameter. That both wild and domestic plants have similar leaf area–stem volume relations raises the question of whether the smaller leaves of domestic plants are associated with different stem length–diameter relationship. Stem length and stem diameter scaled similarly in domestic and wild plants (nonsignificant log_10_ stem diameter cultivated/wild interaction, *p* = .769; Table 1), but with different intercepts (*p* = .043; Table 1). Wild plants had longer stems for a given diameter than domestic plants (Figure 2d). Although not mirrored in the fixed component of models, the random component suggested different slopes and intercepts for the different populations/varieties (Table S4). The >1 slope observed (Table 1) is typical for small plants in active growth (Niklas, 1995).

### 3.4 Stem biomechanics

Differences in the proportions of any biological structure will result in differences in mechanical behavior, so given the differences between domestic and wild poinsettias, we tested for differences in stem resistance to bending. Across the 85 segments tested, segments ranged in diameter from 5.0 to 8.3 mm in domestic plants, and from 4.4 to 10.6 in wild stems. Segments from domestic populations had distances from the tip ranging 21 to 42 cm, whereas the segments from wild populations were 25 to 88 cm from the tips of stems, reflecting the longer stems of wild plants. *E*_stem_ ranged widely, from 195.1 to 1,600 MPa in segments from domestic individuals, and 297.2 to 2,834.0 MPa in those from wild stems. The model examining *E*_stem_ between domestic and wild plants had a nonsignificant interaction term (*p* = .126), indicating similar log_10_ *E*_stem_ ‐ log_10_ distance from the stem tip scaling slopes between cultivated and wild plants. We refit the model without the interaction term, finding that the domestic/wild binary variable was also not significant (*p* = .796), indicating no differences mean *E*_stem_ for a given distance from the tip between wild and domesticated poinsettias (Figure 2e). These results show that domestic and wild poinsettias do not differ in their tissue level mechanical properties, that is, are made of “material” of the same resistance to bending. Given that domestic poinsettias have a lower stem length–diameter intercept than wild plants, a segment of a given length of stem will be more resistant to bending in the domestic as compared to the wild plants by virtue of stem dimensions and not because they have stiffer tissues.

## 4 DISCUSSION

Our data clearly rejected our expectation that domesticated poinsettia plants should have greater leaf area controlling for stem volume than wild plants. Despite the sustained and explicit efforts of breeders for 200 years along vectors of selection that would eagerly identify, preserve, and enhance variants with more leaf area per unit stem volume than the wild plants, in the cultivars we examined, no such tendency was detectable. Instead, the denser outline of cultivated plants is achieved in part via shorter internode length, together with smaller individual leaf area and thicker stems. We discuss how artificial selection on domestic poinsettias has led to manifold changes in proportionalities between parts, all in the context of a constant leaf area–stem volume relationship, and comment on why this relationship is unlikely to be overcome even under artificial selection.

The leaf area–stem size relation is nearly pervasive, being found across virtually all species that have been studied (Ackerly, 1996; Ackerly & Donoghue, 1998; Leslie et al., 2014; Sun et al., 2006; Westoby et al., 2002; White, 1983a,b; Wright et al., 2006). The only exceptions seem to be certain plants in warm drylands, which have greatly reduced leaf area for a given stem size (Eggli, 2002). For example, species such as *Adenium socotranum* have crowns of very small leaf areas but very large stem diameters. Species such as *Alluaudia procera* or *Fouquieria purpusii*, which have thick, spiny stems clothed with small leaves, would also seem to be candidates that depart from the common leaf area–stem volume relationship. For their part, cacti and other stem succulents are often entirely leafless. These examples all come from situations in which productivity is low and selection favors storage, and suggest that it is possible for organs largely dedicated to storage and that are thus not metabolically highly demanding, to survive with relatively low leaf areas. These examples also suggest that these highly water‐storing plants likely have stem tissues of relatively low metabolic rate, a prediction consistent with the few data available, which show that woods with greater storage capacity are less metabolically active (e.g., Lambers, Scheurwater, Mata, & Nagel, 1998). These departures all are in the direction of less rather than more leaf area for a given stem size. That domestic poinsettias also do not move into leaf area–stem volume scaling space that corresponds to “too much” leaf area suggests that while stem tissue can be largely devoted to storage and has low metabolic demands, leaves are a different matter. It suggests that as the sites of photosynthesis, which require water and mechanical support, leaf metabolic demands are necessarily relatively high and cannot in general, as in the case of storage stems, be significantly reduced. Storage stems provide examples of reduced metabolic demands permitting reduced leaf areas; a given amount of standard nonsucculent leaf, however, seems to require a given amount of stem volume to meet its metabolic needs. The mutual metabolic balance between stems and leaves, with stems having a much wider envelope of possible metabolic rates than leaves do with regard to their lower limits, would thus explain why the “too little” leaf area space is occupiable, albeit sparsely, whereas the “too much” leaf area is apparently not accessible, even, as in the case of the domestic poinsettia, under conditions of selection that would reasonably be expected to favor greater leaf area. The readily testable prediction that emerges from our results is therefore that metabolic rate should vary markedly in stems across species (being especially low in water‐storing pachycauls with reduced leaf area; see, e.g., Poorter, Remkes, & Lambers, 1990; Poorter & Remkes, 1990; Lambers et al., 1998) but much less in leaves across plant functional types, from conventional plants to xerophytes with reduced leaf area and stems given over to storage (cf. Jin, Dai, Sun, & Sun, 2008).

That selection strongly maintains the leaf area–stem volume relationship means that selection on any aspect of vegetative allometry necessarily results in predictable changes in subsequent generations (Figure 2). In the case of the poinsettia, selection favoring fuller outlines has resulted in shorter internodes. Shorter internodes clearly achieve the goal of fuller outlines, as compared to the wild plants. Internodes in the wild plants we measured could reach nearly 10 cm, which would lead to a sparse appearance in a small potted plant. Artificial selection has greatly reduced internode differences, with domestic plants having a mean internode distance of just 14.7 mm, as compared to the significantly longer mean of 47.8 mm in wild plants. Reducing internode length in the context of a constant leaf area–stem volume relationship would result in more leaves per unit stem length. More leaves for a constant leaf area necessarily requires smaller individual leaf area, and this is exactly what we observed: domesticated poinsettias have markedly smaller leaves for a given stem volume than wild ones (Figure 2b). Given an apparently insuperable (or at least very strongly favored) leaf area–stem volume relationship, selection for fuller outlines via shorter internodes has thus necessarily involved a reduction in individual leaf area.

In addition to affecting individual leaf area, a constant leaf area–stem volume relationship means that selection for fuller plant outlines could in principle also affect stem length–diameter proportions. Shorter stems for a given diameter (or thicker stems for a given length) would result in greater stem volume per unit length. Given a constant leaf area–stem volume relationship, greater stem volume per unit length necessarily offers greater opportunities per unit length for the deployment of leaf area. As a result, it is entirely plausible that selection favoring fuller outlines in domestic poinsettias could alone account for the thicker stems of domestic poinsettias as compared to wild ones of similar heights **(**Figure 2d**).**

Also potentially accounting for the greater thickness of domestic poinsettia stems is that another stated goal of poinsettia breeders is to increase breakage resistance (Taylor et al., 2011). Shipping poinsettias to market involves mechanical loads that can deform or break branches, leading to loss of revenue. When cultivars are presented to growers, among variables such as bract color, plant size, and flowering time, the resistance to stem mechanical deformation is also routinely reported (Ecke, 2011). Our results show that domestic poinsettia stems are indeed more resistant to bending but suggest that breeding efforts have not achieved these more resistant stems via greater materials stiffness, as reflected by *E*_stem_, but through thicker stems. The stiffness of a stem depends on two variables, the capacity of the material it is made up of to resist bending (as reflected by *E*_stem_)*,* and how well the material in a given stems is deployed in space to resist bending (as reflected by *I*, the second moment of area, Niklas, 1992). That *E*_stem_ does not vary between domestic and wild poinsettias (Figure 2e), but that domestic plants have thicker stems (Figure 2d), allowed us to identify *I*, which scales with stem radius to the fourth power, as the crucial variable contributing to differences in resistance to bending between wild and domestic poinsettias. Selection favoring resistance to bending could thus plausibly have led to the thicker stems of domestic poinsettias. As mentioned above, thicker stems for a given length mean greater opportunities for deployment of leaf area, and therefore fuller outlines. As a result, selection favoring increase in leaf area could also increase stem volume for a given stem length. Whatever the exact cause, it illustrates how the constant leaf area–stem volume relationship produces a ripple effect, with any one change in the value of one of the variables making up part of the allometric network leading to predicable changes elsewhere (Figure 2).

Such phenomena are often discussed under the rubric of “constraint,” but we suggest that there is little to be gained by use of the term. The leaf area–stem volume relationship being “fixed” does indeed mean that changes in other traits such as leaf area, stem length–diameter proportions, or internode distances, lead to changes in the other traits that are predictable precisely because of the “fixed” leaf area–stem volume relationship. This predictability diagnoses limited or biased outcomes, exactly the notion that many definitions of constraint aim to capture (Olson, 2012). However, it seems likely that the leaf area–stem volume relationship is not “fixed” in any way beyond simply being one strongly favored by selection from among a range of developmentally possible contenders. That the “too little” leaf area condition is possible to observe in nature shows vividly that such variants can be produced. Although the poinsettia does not provide an example of an occupant of the “too much” leaf area zone, there are other ways other than artificial selection of testing whether this area is accessible or not and the causes of this (in)accessibility. A comparative approach is the prediction that leaf metabolic rate should vary less across species than in the stem. An experimental approach could examine our prediction that the leaf area–stem volume relationship is maintained because of the strong metabolic interdependence between stem and leaf, implying that leaves require a certain amount of stem volume (Donovan, Maherali, Caruso, Huber, & de Kroon, 2011; Olson, 2012). Experimental removal of stem tissue would help quantify the functional and fitness impacts of having “too much” leaf for a given stem volume. From this point of view, the leaf area–stem volume relationship is subject to stronger selection than others, including the total leaf area–leaf size relationship and stem length–diameter allometry. Although such situations are often described as representing “allometric,” “selective,” or “adaptive” constraint, use of these terms does not in fact add any mechanistic or detail or predictive power and we would advocate avoiding the vague term “constraint” in favor of describing the mechanism leading to any restriction or biasing of outcomes in as much detail as possible (Olson, 2012).

The apparently very strong selection favoring a constant leaf area–stem volume relationship implies that many trait combinations are unlikely to be achieved, both in nature and in the domestic poinsettia. That the combination of high leaf area and thin stems is not readily available seems underscored by horticultural practice in the poinsettia industry. In the apparent absence of breakage‐resistant variants with greater leaf area per unit stem diameter, full and rounded outlines in domesticated poinsettias are achieved by pinching, which involves manual removal of apical meristems, thereby encouraging branching (Kobayashi, 2012; Lee et al., 1997), and even infection with phytoplasma, which also has the effect of encouraging branching (Lee et al., 1997). That poinsettia horticulturalists employ these labor‐intensive and expensive techniques to produce plants of the requisite proportionalities strongly suggests that the desired increase in leaf area is not feasible and needs to be achieved through branching, illustrating how the mapping of patterns of covariation can guide applied breeding efforts in addition to shedding light on adaptive processes in nature.

## 5 CONCLUSION

Our data show that two centuries of selection on the Christmas poinsettia have altered neither the slope nor the intercept of the leaf area–stem volume relationship. The apparent fixity of this relationship means that selection favoring fuller outlines during the domestication of the poinsettia has favored shorter internodes, leading to branches more densely clothed with leaves. Shorter internodes require more leaves per unit stem length, and given a constant leaf area–stem volume relationship, more leaves necessarily require smaller leaves. At the same time, stem length–diameter relations have been altered, with domestic poinsettias having thicker stems, leading both to more breakage‐resistant stems as well as shorter internodes. With elaborate pinching and phytoplasma infection practices, the poinsettia provides a vivid example of the expense and effort required to produce desired morphologies when the path to their breeding is blocked by powerful selection favoring the leaf area–stem volume relationship above all others.

## CONFLICT OF INTEREST

None declared.

## DATA ARCHIVING

Data are available in Tables [Link], [Link], [Link].
