# Supplementary material for: Nearly 200 years of sustained selection have not overcome the leaf area–stem size relationship in the poinsettia
Source: Evol Appl. 2018 May 16;11(8):1401–11. doi: 10.1111/eva.12634 (PMC6099819; doi:10.1111/eva.12634)
Supplement: Supplementary file 4 [file EVA-11-1401-s004.docx]

Table S4. Mixed effects models fit to test for differences in the allometry of cultivated and wild poinsettias. We compared the fit of random intercept models vs. random slope & random intercept models through likelihood ratio tests (LRT) and the Akaike Information Criterion (AIC). In bold, random component in model that best fit the data.

| **Model** | **Random component in model** | **LRT** | **AIC** |
| --- | --- | --- | --- |
| Total leaf area ~ stem volume + cultivated/wild  (Branches/individuals within populations/varieties) | **Random intercept** | P = 0.999 | **-61.4** |
|  | Random slope & intercept |  | -57.4 |
| Mean leaf area ~ stem volume + cultivated/wild  (Branches/individuals within populations/varieties) | **Random intercept** | P = 0.340 | **-123.35** |
|  | Random slope & intercept |  | -121.51 |
| Internode distance ~ stem volume + cultivated/wild  (Branches/individuals within populations/varieties) | **Random intercept** | P = 0.265 | **-213.27** |
|  | Random slope & intercept |  | -211.93 |
| Stem length ~ stem diameter + cultivated/wild  (Branches/individuals within populations/varieties) | Random intercept | P = 0.012 | -87.85 |
|  | **Random slope & intercept** |  | **-92.71** |
| *E_stem_* ~ distance to the tip + cultivated/wild  (Segments within individuals; individuals within populations/varieties) | **Random intercept** | P = 0.967 | **-12.84** |
|  | Random slope & intercept |  | -5.41 |
